# Orientation-Controllable ZnO Nanorod Array Using Imprinting Method for Maximum Light Utilization in Dye-Sensitized Solar Cells

**DOI:** 10.1186/s11671-015-0961-9

**Published:** 2015-06-12

**Authors:** Huisu Jeong, Hui Song, Ryeri Lee, Yusin Pak, Yogeenth Kumaresan, Heon Lee, Gun Young Jung

**Affiliations:** School of Materials Science and Engineering, Gwangju Institute of Science and Technology (GIST), Gwangju, 500-712 Republic of Korea; Department of Materials Science and Engineering, Korea University, Seoul, 136-701 Republic of Korea

**Keywords:** Dye-sensitized solar cell, TiO_2_ patterning, Scattering layer, ZnO nanorods, Light harvesting

## Abstract

We present a holey titanium dioxide (TiO_2_) film combined with a periodically aligned ZnO nanorod layer (ZNL) for maximum light utilization in dye-sensitized solar cells (DSCs). Both the holey TiO_2_ film and the ZNL were simultaneously fabricated by imprint technique with a mold having vertically aligned ZnO nanorod (NR) array, which was transferred to the TiO_2_ film after imprinting. The orientation of the transferred ZNL such as laid, tilted, and standing ZnO NRs was dependent on the pitch and height of the ZnO NRs of the mold. The photoanode composed of the holey TiO_2_ film with the ZNL synergistically utilized the sunlight due to enhanced light scattering and absorption. The best power conversion efficiency of 8.5 % was achieved from the DSC with the standing ZNL, which represented a 33 % improvement compared to the reference cell with a planar TiO_2_.

**Electronic supplementary material:**

The online version of this article (doi:10.1186/s11671-015-0961-9) contains supplementary material, which is available to authorized users.

## Background

The dye-sensitized solar cell (DSC) is a promising daylight-harvesting appliance because of its simple and low-cost fabrication, eco-friendly manufacturing, color modulation, and suitable building integration. The conventional DSC consists of a dye-adsorbed photoanode, electrolyte, and counter electrode, among which the photoanode is the key element for obtaining high photocurrents and high power conversion efficiency (PCE). A film of titanium dioxide (TiO_2_) nanoparticles with a diameter of less than 15 nm is usually used for the photoanode to adsorb more dyes, but the poor usage of incident light because of a high transparency of 20 ~ 60 % at visible wavelengths is troublesome. A thick TiO_2_ nanoparticulate film allows lower transmittance but induces a long charge diffusion length, which results in a higher probability of electron recombination during migration.

Currently, there are two approaches to recycle more incident light by modifying the planar TiO_2_ nanoparticulate photoanode. The first approach is to pattern the TiO_2_ film by various lithographic methods such as soft imprinting [1], polystyrene sphere templating [2–4], 3-dimensional holography [5], and glass texturing [6, 7]. The patterned TiO_2_ films allow more incident light to recycle inside the photoanode for enhanced photocurrents and PCE. The second approach is adding a scattering layer on top of the TiO_2_ nanoparticulate film. Generally, micron- or submicron-scaled metal oxide crystals are introduced in the form of spheres [8–12], rods [13–15], prisms [16, 17], sheets [18], cubes [19], tubes [20], and flower clusters [21] as the scattering layer.

Recently, maximum utilization of the incident light was achieved by combining both methods above: the patterned TiO_2_ film with the scattering layer on top. For example, Char et al. [22] introduced a micro-pyramidal TiO_2_ photoanode coated with a TiO_2_ scattering particulate film, which exhibited a higher PCE than the solitarily patterned TiO_2_ film. In addition, Moon et al. [23] reported a TiO_2_ nanorod (NR)-planted 3-dimensional inverse opal TiO_2_ film, where the NRs were incorporated as additional scattering media.

In this study, we propose a novel fabrication method to obtain a patterned TiO_2_ nanoparticulate film combined with an additional scattering layer simultaneously by combinatorial techniques of imprinting and transfer method. A periodically aligned vertical ZnO NR array was used as an imprint mold to pattern the TiO_2_ nanoparticulate film, and the ZnO NR array was transferred onto the TiO_2_ film as a light scattering and absorbing layer while imprinting. Therefore, patterning the TiO_2_ film and transferring the ZnO NR layer (ZNL) were accomplished concurrently. Furthermore, the orientation of ZNL could be controlled by altering the ZnO NR configuration on the mold, such as the pitch, size, and height.

## Methods

### Fabrication of ZnO NR Mold

A mold with periodically aligned vertical ZnO NRs on a GaN substrate was achieved by polymer-templated hydrothermal growth [24, 25]. The GaN substrate is appropriate for vertical ZnO NR growth because of the well-matched epitaxial growth between GaN and ZnO [24]. A 10-nm-thick ZnO film was deposited by sputtering onto the GaN substrate as a seed layer, and the polymer hole template was obtained by nanoimprint lithography. An UV-curable imprint resin composed of polydimethylsiloxane material (Gelest), ethylene glycol dimethacrylate (Aldrich), and Irgacure 184 (Ciba) was prepared with a weight ratio of 87:10:3, respectively. This nanoimprint resin was spin-coated at 6000 rpm for 200 s on the ZnO seed/GaN substrate and subsequently UV-imprinted using a transparent stamp with periodic nanopillars at 7 bar for 8 min. After detaching the stamp from the UV-cured polymer resin, a dry etching process was performed using a CF_4_ gas plasma (50 sccm, 20 mTorr, 20 W, 30 s) to remove any residual layer under the hole trenches until the underlying ZnO seed layer was exposed.

Next, the ZnO NRs were grown by hydrothermal process. The polymer-templated GaN substrate was immersed into a prepared nutrient solution, which consisted of zinc nitrate hexahydrate (Zn(NO_3_)_2_ · 6H_2_O) and hexamethylenetetramine (C_6_H_12_N_4_) (both had concentrations of 5 mM in DI water) [24]. The ZnO NR growth was proceeded in an oven at 92 °C, and the growth time was determined based on the ZnO NR height. The fabrication scheme and relevant scanning electron microscope (SEM) images are illustrated for easy understanding in Additional file 1: Figure S1.

### DSC Fabrication

A TiO_2_ nanoparticulate paste was prepared by the previously reported recipe and doctor-bladed on a cleaned fluorine-doped tin oxide (FTO) glass (Pilkington, TEC-7, 8 Ω sq^−1^) [26]. A semi-dried TiO_2_ film was prepared by baking on a hot plate at 80 °C for 30 min. After imprinting and transferring the ZnO NRs, the sample was fully sintered at 450 °C for 30 min under air condition. The photoanodes were finalized by immersing into a solution of N719 dye (0.3 mM in ethanol) for 3 h at 80 °C [27]. The dye-adsorbed photoanode was assembled with a catalytic platinum counter electrode at a gap of 30 μm using a Surlyn film (Dupont), which was filled later with an electrolyte (Solaronix, Iodolyte AN-50).

## Results and Discussion

**Fig. 1:**
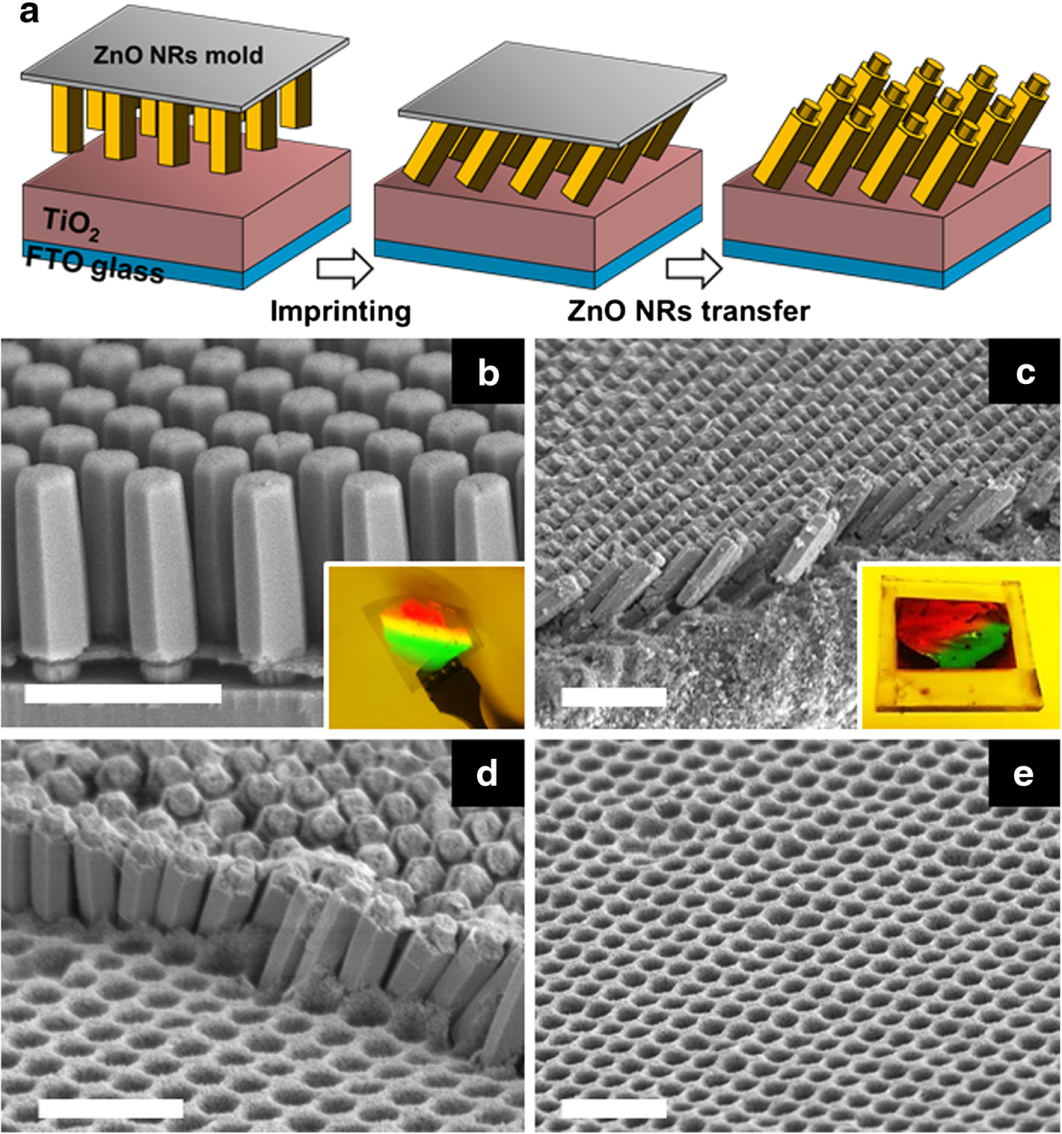
**a** Schematic of photoanode fabrication: simultaneous process of patterning a TiO_2_ film and transferring the ZnO NRs while imprinting. SEM images: **b** the mold with periodically aligned *vertical* ZnO NRs on the GaN substrate. The *inset* is a photo image of the whole ZnO NR mold (a quarter size of 2-in. GaN wafer). **c** The transferred tilted ZnO NR array on the TiO_2_ film. The *inset* shows a dye-adsorbed ZNL/TiO_2_ photoanode in an area of 4 cm^2^. **d** A defect area, where ZnO NRs were not transferred on the TiO_2_ film, showing hole trenches. **e** The holey TiO_2_ film, which was achieved by complete wet etching of the embedded ZnO NRs with an acidic solution. All *scale bars*, 2 μm

Figure 1a is the schematic image of the fabrication process for the patterned TiO_2_ film with the ZNL on top. The grown ZnO NR has dual diameters of 250 nm (root part) and 500 nm (trunk part) as shown in the SEM image of Fig. 1b. The hole in the polymer template determined the root diameter of the NR, and the diameter of the ZnO NR above the hole was horizontally expanded with the growth. The ZnO NR array with a height of 2 μm at 800-nm pitch occupied an area of 4 cm^2^ (inset of Fig. 1b). The TiO_2_ film was prepared with TiO_2_ nanoparticulate paste by doctor blading method on a FTO glass, and the sample was baked at 80 °C for 30 min to obtain a semi-dried film instead of a fully dried film. The semi-dried TiO_2_ film was soft enough for being imprinted while still having an adhesive property strong enough to hold the transferred ZnO NRs during imprinting. In addition, the ZnO NR mold was treated with an AZ 100 solution prior to imprinting, which weakened the connection of the ZnO NRs to the GaN substrate, thereby inducing the easy separation of the ZnO NRs from the substrate during demolding (Additional file 1: Figure S2).

The imprint process between the ZnO NR mold and the semi-dried TiO_2_ film was performed using an embossing machine at 10 bar for 15 s. After detaching the mold, the well-aligned ZnO NRs were successfully transferred on top of the semi-dried TiO_2_ film, and then the photoanode substrate was completely sintered at 450 °C for 30 min to remove the remaining organic binders. Figure 1c represents the transferred ZnO NRs on the TiO_2_ film. Highly ordered ZnO NRs are tilted and lean on each other, and the root part of the ZnO NRs is facing upward after transferring. A dye-adsorbed ZNL/TiO_2_ photoanode is shown in the inset of Fig. 1c, which was made in an area of 4 cm^2^ on a FTO glass. Iridescent colors (red-green) appear because of light reflection at the periodic ZnO NR array surface, which demonstrates a faithful transfer of the ZNL uniformly over the entire area. Figure 1d reveals an area where the ZnO NRs were not transferred onto the TiO_2_ film defectively. Imprinted holes by the ZnO NRs appeared on the TiO_2_ film, in which the ZnO NRs should be embedded if properly transferred. A holey TiO_2_ film without the ZNL was also possible after removing the ZNL by chemical wet etching using a hydrochloric acid solution before dye adsorption, as shown in Fig. 1e.

**Fig. 2:**
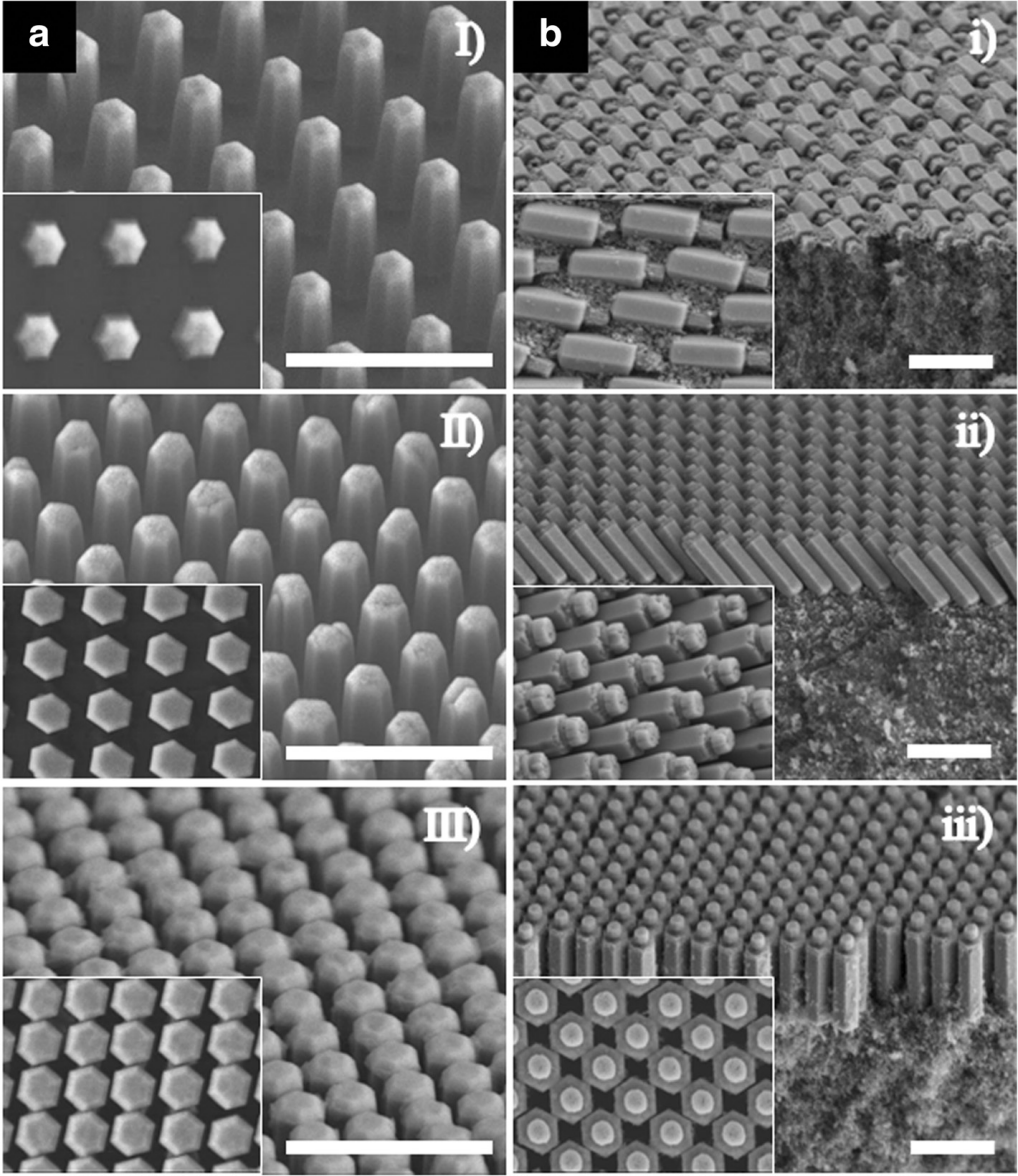
**a** SEM images of three types of ZnO NR molds with different heights and pitches: (*I*) 1.2 μm and 1 μm, (*II*) 2 μm and 800 nm, (*III*) 2 μm and 650 nm. **b** (*i*)–(*iii*) The ZNLs on a nanoparticulated TiO_2_ film after imprinting using a mold that corresponds to (*I*)–(*III*), respectively. The SEM images of the transferred ZNL in a large area are supplied in Additional file 1: Figure S4. All *insets* show the *top view* of ZnO NRs in the area of 2.4 × 3.2 μm^2^. All *scale bars*, 2 μm

Interestingly, the ZnO NRs were transferred with different orientations onto the TiO_2_ film using diverse ZnO NR molds, on which the NRs had different heights and pitches. Figure 2a is a set of SEM images of ZnO NR molds. The height of the ZnO NR was determined based on the growth time, and the pitch was varied depending on the polymer template (Additional file 1: Figure S3). Three types of ZnO NR molds with different heights and pitches were used: (I) 1.2 μm and 1 μm, (II) 2 μm and 800 nm (the same as Fig. 1b), (III) 2 μm and 650 nm. The diameter of the ZnO NR was 500 nm in all molds, as illustrated in the insets. Figure 2b(*i*)–(*iii*) shows the images of the transferred ZnO NRs with the corresponding molds of Fig. 2a(*I*)–(*III*), respectively. The produced ZNL had three different orientations: (i) laid, (ii) tilted, and (iii) standing states, illustrating that the orientation of ZnO NRs on the TiO_2_ film was determined by the ZnO NR configuration on the mold. In comparison to the ZnO NRs (II), the ZnO NRs (I) were likely to lie down completely on the TiO_2_ film after imprinting because of their short height and the adequate space between the NRs. On the contrary, the ZnO NRs (III) were difficult to collapse during imprinting because they were nearly in contact with the neighboring ZnO NRs. We prepared five different photoanodes, including the three types of ZNL cells, a planar TiO_2_ film, and a holey TiO_2_ film (Fig. 1e), to figure out the effect of ZNL and ZnO NR orientation on the photovoltaic properties.

**Fig. 3:**
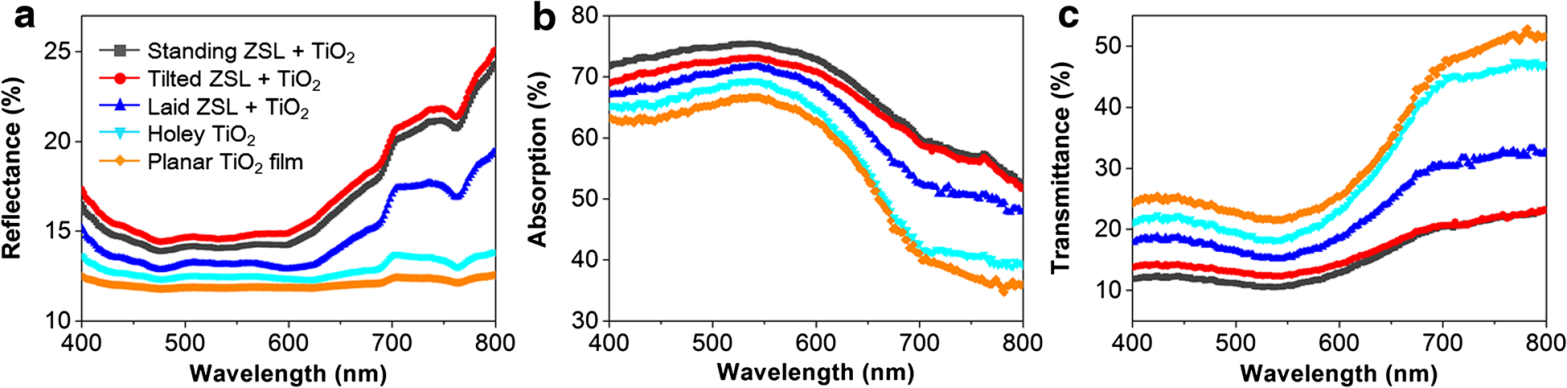
Optical characteristics of the five different photoanodes after dye adsorption. **a** Reflectance, **b** absorption, and **c** transmittance

Generally, with more dye molecules adsorbed onto the photoanode, more photo-generated electrons can be harvested. Therefore, to exactly compare the light utilization within the five photoanodes, the amount of dye loading in every cell should be identical. Because the ZnO NRs could also adsorb dye molecules, the TiO_2_ nanoparticulate film of the two reference cells (planar TiO_2_ and holey TiO_2_ films) without the ZNL was of 13 μm, which was 1.5 μm thicker than that of the ZNL photoanodes, considering the dye adsorbability of ZnO NRs. To verify the amount of loaded dye, the dye-adsorbed photoanodes were immersed into a 1 mM KOH aqueous solution for 2 h to completely desorb the dye molecules. The absorbance spectra of the desorbed dye solutions revealed that the amount of adsorbed dye molecules was nearly identical in the five photoanodes (Additional file 1: Figure S5). Optical characteristics such as reflectance (*R*) and transmittance (*T*) were measured with the dye-loaded photoanodes in the wavelength range of 400–800 nm with an illumination to the FTO glass face. The absorption (*A*) spectrum was calculated using the following equation: *A* = 100 (%) − *R* − *T*. In Fig. 3a, the holey TiO_2_ film shows a slightly higher reflectance than the planar one because of the patterning of the TiO_2_ film, and the ZNL photoanodes show a much higher reflectance than the holey TiO_2_ film in the entire visible range because of the additional scattering effect from the ZNL.

The best light-scattering effect appeared in the tilted ZNL photoanode because the tilted NRs occupied the entire TiO_2_ film tightly without any empty space, as shown in the inset of Fig. 2b(*ii*). The closely packed tilted ZnO NR array works better as a light-scattering layer than the laid and standing ZNLs, which have voids between the neighboring NRs as shown in the insets of Fig. 2b(*i*) and (*iii*), respectively. However, in terms of light absorption (Fig. 3b), the standing ZNL photoanode exhibits the best light absorbance because the vertically standing dye-adsorbed ZnO NRs can trap more incident light than those in tilted state (see Additional file 1: Figure S6) [28]. Since more efficient light utilization can be achieved by absorbing and scattering more light within the photoanode, a lower transmittance of the photoanode is a prerequisite for high-efficiency DSCs, which can be adjusted by controlling the orientation of the transferred ZnO NRs in this experiment. Overall, the standing ZNL photoanode has the lowest transmittance in the entire visible range (Fig. 3c).

**Fig. 4:**
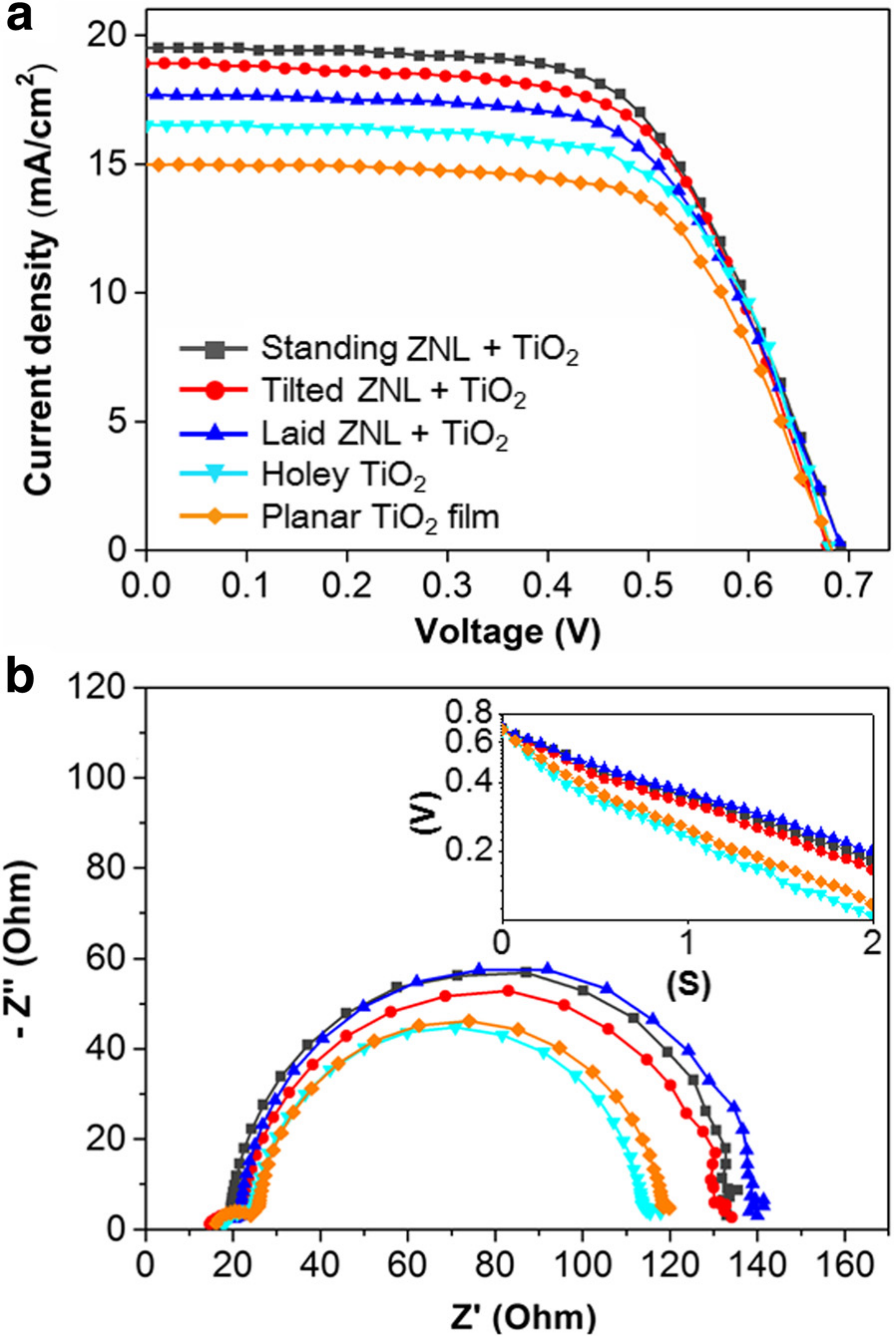
**a** Photocurrent density versus voltage characteristics and **b** Nyquist plots of the DSCs with different photoanode configurations. The EIS measurement was performed under the dark at −0.7 V. The *inset* shows open-circuit voltage decay *curves* right after turning off illumination

Figure 4a shows the photocurrent density (*J*)–voltage (*V*) curves of the front-side illuminated DSCs with the five different photoanodes. Table 1 summarizes the photovoltaic properties of these cells. All DSCs were characterized using a Keithley 2400 source meter under air mass (AM) 1.5 simulated sunlight (100 W∙cm^−2^ intensity). An identical active area of 0.25 cm^2^ was exposed using a shadow mask. The holey TiO_2_ cell revealed a *J*_sc_ value of 16.6 mA∙cm^−2^ and a PCE value of 7.2 %, which were higher than 14.9 mA∙cm^−2^ and 6.4 % from the planar TiO_2_ one, respectively. The DSCs with the ZNL were superior to the holey TiO_2_ DSC in both *J*_sc_ and PCE, which indicates that more photo-exited electrons were generated. The highest *J*_sc_ and PCE (19.4 mA∙cm^−2^ and 8.5 %) were obtained with the standing ZNL photoanode. It is noteworthy that the *J*_sc_ and PCE values increased inversely with the transmittance (Fig. 3c), indicating more efficient light recycling and utilization at lower transmittance.

**Table 1:** Photovoltaic properties of the DSCs with different photoanodes

	*J*_sc_ (mA cm^−2^)	*V*_oc_ (V)	FF	PCE (%)
Planar TiO_2_	14.9	0.68	0.63	6.4
Holey TiO_2_	16.6	0.68	0.64	7.2
Laid ZNL + TiO_2_	17.7	0.69	0.65	7.9
Tilted ZNL + TiO_2_	18.9	0.68	0.64	8.2
Standing ZNL + TiO_2_	19.4	0.69	0.64	8.5

An electrochemical impedance spectroscopy (EIS) analysis was performed to understand the charge transfer resistance in the different photoanodes. Figure 4b presents the Nyquist plots of the five DSCs in the frequency range of 10^5^–10^−1^ Hz under the dark at −0.7 V. Normally, three semi-circles appear in the Nyquist plots from the left to the right with decreasing frequencies, which correspond to the reduction at the counter electrode (10^5^–10^3^ Hz range), electron recombination resistance at the photoanode (10^3^–1 Hz range), and Nernst diffusion in the electrolyte (1–10^−1^ Hz range) [29]. However, in this study, only two semi-circles appeared because the electrolyte impedance was overlapped with the electron recombination resistance of the photoanode due to the relatively fast electron diffusion through the 30-μm-gap-distance electrolyte [30]. The second semi-circle expanded when the ZNL was applied, demonstrating that the recombination resistance of the photoanode increased [30–32]. The higher recombination resistance signifies that the photo-generated electrons can diffuse for a longer time prior to trapping or charge recombination. Thinner nanoparticulate TiO_2_ films [33, 34] and ZnO NR-embedded TiO_2_ film [30] can lead to lower surface state traps and thus a lower probability of electron recombination. As the cells with the ZNL have a thinner TiO_2_ film along with the ZnO NRs on top, they are beneficial for reducing the electron recombination.

The lifetime of the DSCs was measured by open-circuit voltage decay (OCVD) method. The DSCs were illuminated at AM 1.5 (100 mW∙cm^−2^), and the generated photovoltage was monitored at 70-ms interval using an oscilloscope (Tektronix, DPO4014B) immediately after turning off the illumination. The OCVD result (inset of Fig. 4b) shows that the ZNL cells have a slower voltage drop than the cells without it, which indicates a longer electron lifetime. The OCVD curves of the ZNL cells are approximately on top of each other. The electron lifetime can be calculated with the OCVD curve according to the following equation [35, 36]:

$$ {\tau}_{\mathrm{e}}=\left(kT/q\right){\left|\left(\mathrm{d}{V}_{\mathrm{oc}}/\mathrm{d}t\right)\right|}^{-1} $$

where *k* is Boltzmann’s constant, *T* is the absolute temperature, *q* is the electron charge, *V*_oc_ is the open-circuit voltage of each cell, and *t* is the time. The calculated electron lifetimes are 39.5 ms (standing ZNL cell), 36.7 ms (tilted ZNL cell), 40.6 ms (laid ZNL cell), 24.3 ms (holey TiO_2_ cell), and 26 ms (planar TiO_2_ cell). These results are consistent with the electron recombination resistance in the Nyquist plots, where the higher recombination resistance signifies the longer electron lifetime. The laid ZNL cell shows a slightly better electron lifetime than the other ZNL cells. However, the superior light utilization (higher light absorption and scattering) of the standing ZNL cell overwhelms the slightly shorter electron lifetime and leads to the highest photovoltaic performance among the cells.

## Conclusions

In summary, periodically transferred ZNLs were successfully fabricated simultaneously onto a TiO_2_ film by imprinting and transferring of ZnO NRs. The orientation of the ZNL could be controlled by the ZnO NR configuration on the mold, such as the pitch and the height. Laid, tilted, and standing ZnO NR-embedded TiO_2_ photoanodes were generated along with a normal flat TiO_2_ film and a holey TiO_2_ film for references. The highest PCE of 8.5 % was achieved from the standing ZnO NR-embedded cell, which represented a 33 % improvement compared to the planar TiO_2_ cell and an 18 % improvement compared to the holey TiO_2_ cell. The improved photovoltaic properties were attributed to the combined effects of superior light utilization and longer electron lifetime, which were evidenced by optical spectroscopy, EIS and OCVD measurements.

## Additional file

Additional file 1: **Electronic supplementary information (ESI).** The file contains supplementary Figure S1–S6.
