# Supplementary material for: Orientation-Controllable ZnO Nanorod Array Using Imprinting Method for Maximum Light Utilization in Dye-Sensitized Solar Cells
Source: Nanoscale Res Lett. 2015 Jun 12;10:263. doi: 10.1186/s11671-015-0961-9 (PMC4472652; doi:10.1186/s11671-015-0961-9)
Supplement: Supplementary file 1 — Electronic supplementary information (ESI). The file contains supplementary Figure S1–S6. [file 11671_2015_961_MOESM1_ESM.docx]

***Electronic Supplementary Information (ESI)***

Orientation-Controllable ZnO Nanorod Array Using Imprinting Method for Maximum Light Utilization in Dye-Sensitized Solar Cells†

Huisu Jeong,^1^ Hui Song,^1^ Ryeri Lee,^1^ Yusin Pak,^1^ Yogeenth Kumaresan,^1^ Heon Lee,^2*^ and Gun Young Jung^1*^

*^1^* School of Materials Science and Engineering, Gwangju Institute of Science and Technology (GIST), Gwangju, 500-712, Republic of Korea.

* Email address: gyjung@gist.ac.kr

*^2^* Department of Materials Science and Engineering, Korea University, Seoul, 136-701, Republic of Korea.

* Email address: heonlee@korea.ac.kr


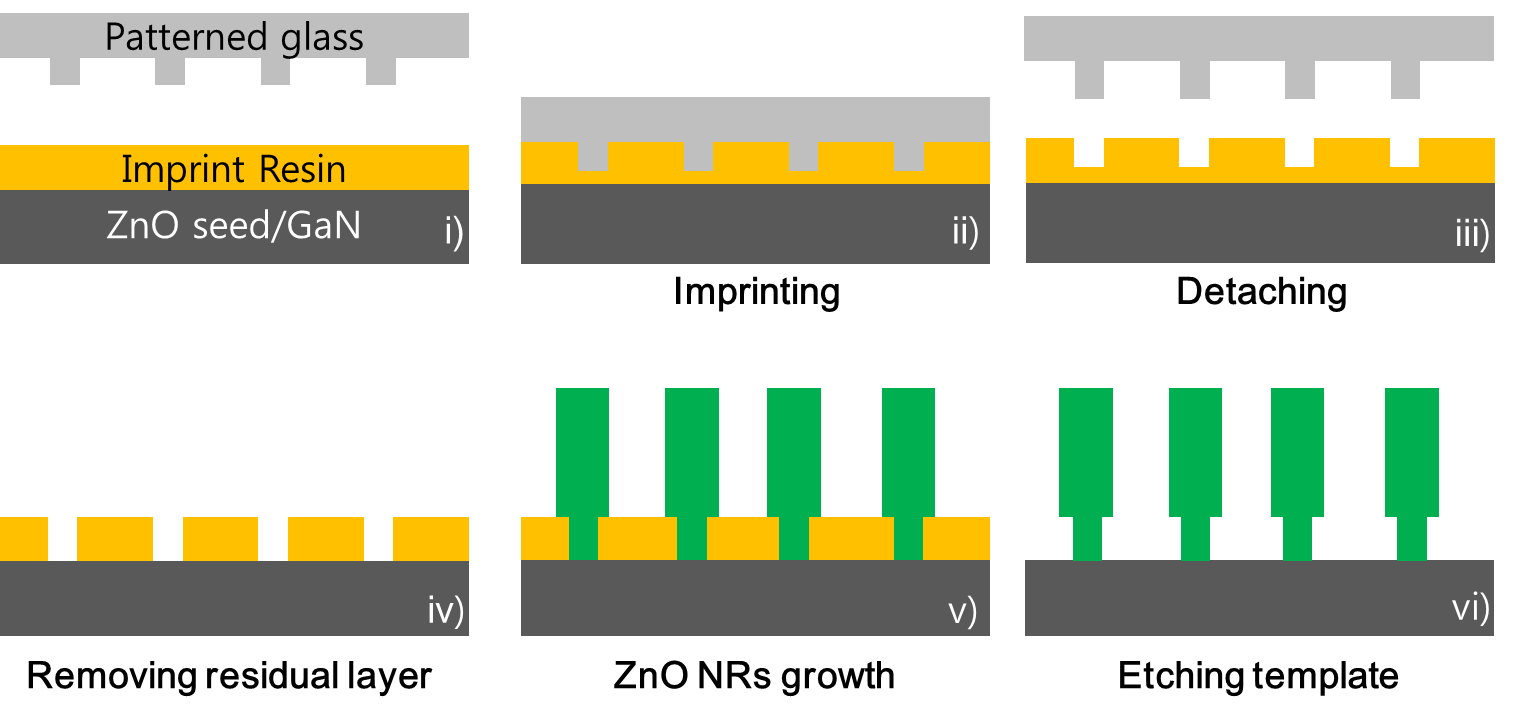


**Figure S1**. **Fabrication of the polymer template and the subsequent ZnO NRs growth.**

Figure S1 shows the fabrication scheme for a ZnO NR mold;

1. Spin-coating of a UV-curable imprint resin on a ZnO seed/GaN substrate.
2. Prior to nanoimprint lithography, a glass stamp with periodic nanopillars is coated with a self-assembled monolayer (1H,1H,2H,2H-perfluoro-decyl-trichloro-silane (FDTS)) for easy demolding. The glass stamp is pressed on the resin and subsequently irradiated with UV light to cure the resin.
3. Detaching the glass stamp from the UV-cured resin.
4. Dry-etching with a CF_4_ gas plasma to remove the residual layer within the hole-trenches and expose the ZnO seed layer underneath.
5. ZnO NRs are grown by hydrothermal method. The ZnO NR has dual-diameters because of the horizontal expansion above the hole.
6. Stripping of the polymer template by oxygen plasma ashing.


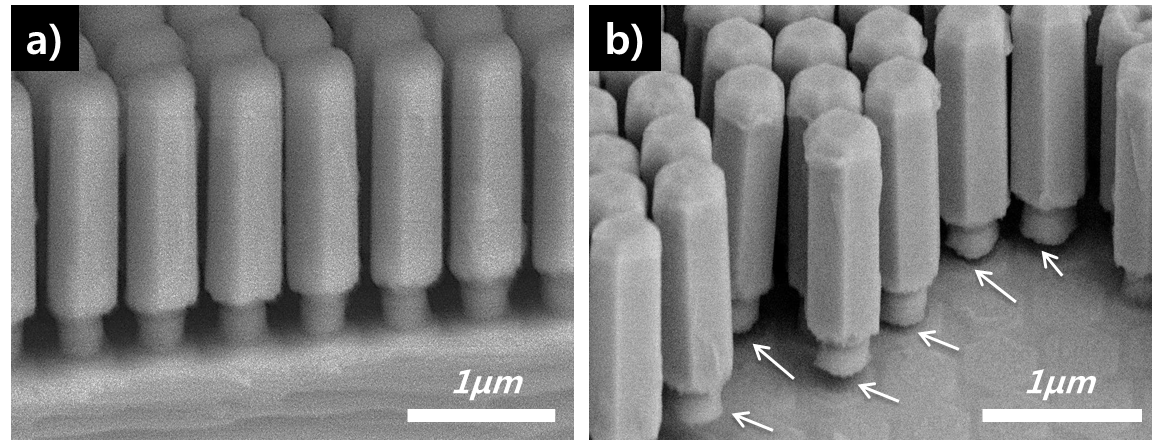
**Figure S2. The AZ 100 treatment for easy separation of ZnO NRs from the mold.**

Figure S2(a) is an SEM image of pristine ZnO NR mold right after hydrothermal growth and polymer template stripping, showing the connection of the ZnO NRs with the GaN substrate through the roots. For easy separation of the ZnO NRs from the substrate in the following imprint step, the ZnO NR mold was immersed in an AZ 100 solution for 60 min at 60 °C prior to imprinting. A basic solution of 1-aminopropan-2-ol (C_3_H_9_NO) in AZ 100 dissolved the ZnO based on the reaction: ZnO + 2OH^-^ + H_2_O → [Zn(OH)_4_]^2-^. Due to a low concentration of hydroxide ions in the AZ 100 (pH = 10.8 at 60 °C), the dissolving reaction was so slow that the ZnO NRs were damaged minimally, but the 10 nm thick ZnO seed was removed during the treatment. Figure S2(b) exhibits the separated ZnO NRs roots from the GaN substrate by the ZnO seed removal (see arrowed position). Therefore, the facile ZnO NRs transfer onto the TiO_2_ film can be possible.


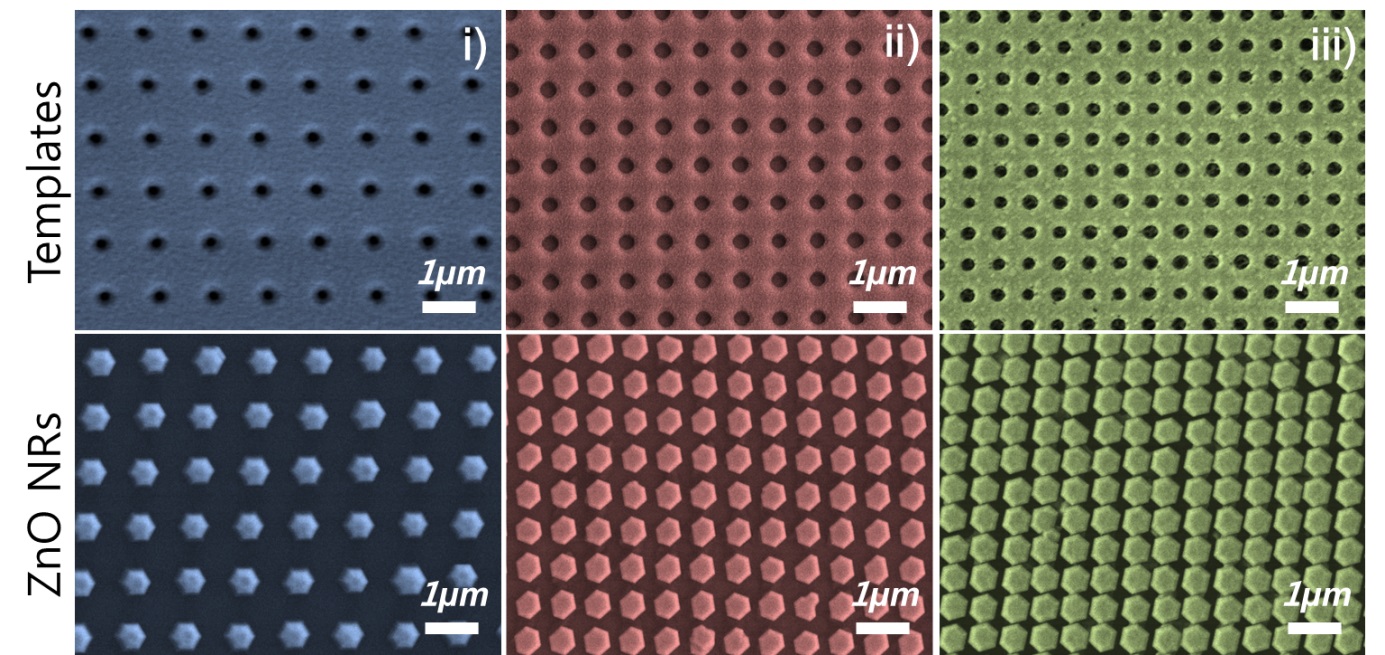


Figure S3. The ZnO NR configurations grown from three different polymer templates.

Figure S3 shows the polymer templates with different pitch sizes and the ZnO NR molds grown from the respective templates. The templates with various pitches were fabricated by nanoimprint technique using glass stamps with nanopillars having different pitch sizes: (i) 1 μm, (ii) 800 nm and (iii) 650 nm. These (i) – (iii) polymer templates were used for the growth of ZnO NRs with different heights, which was possible by controlling the growth time for 60 min for (i) and 90 min for (ii, iii) molds. These molds revealed different ZnO NR orientations in the ZNL after imprinting; i.e., laid-, tilted-, and standing states in order.


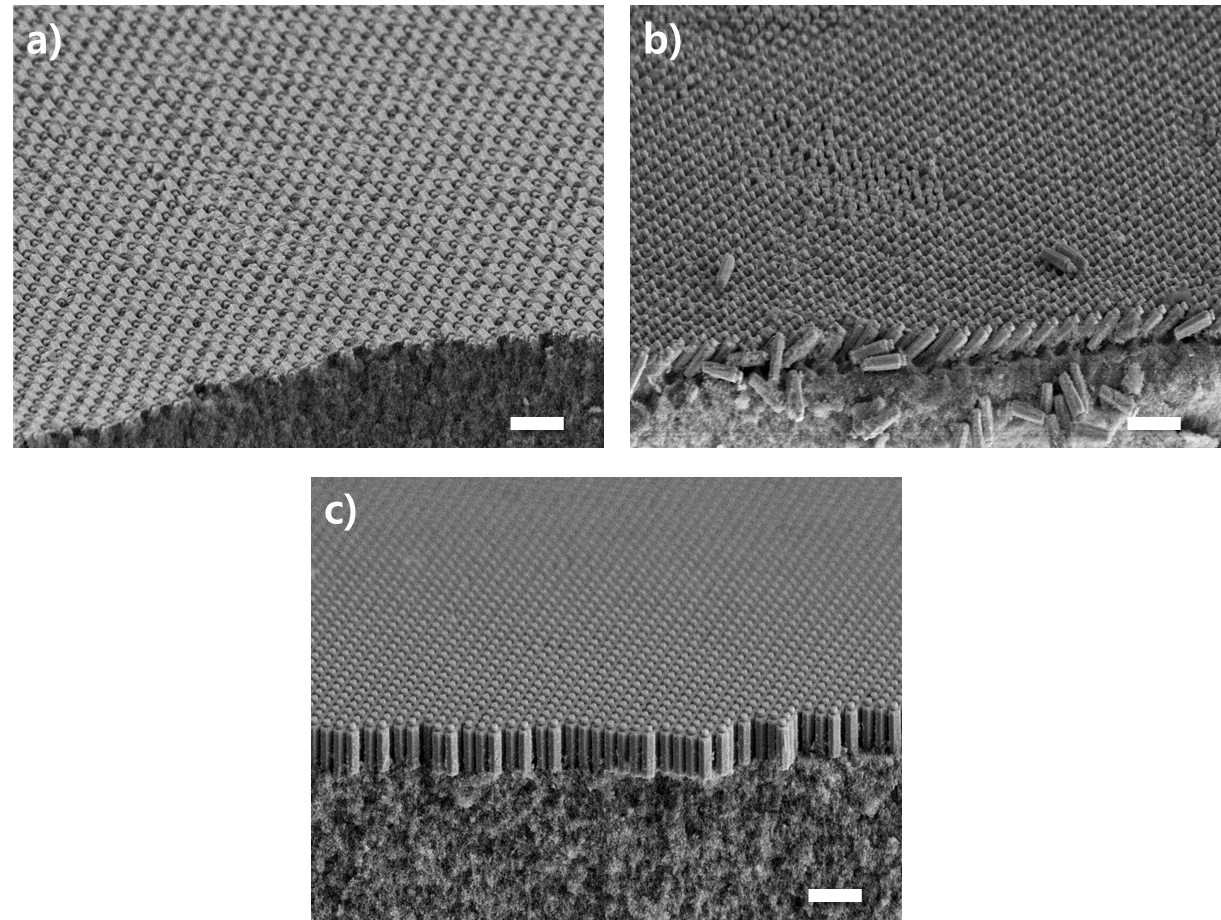


Figure S4. Supplementary SEM images

Figure S4 shows SEM images of the ZNL with (a) laid-, (b) tilted- and (c) standing-ZnO NRs in a large area. (All scale bars: 2 μm)


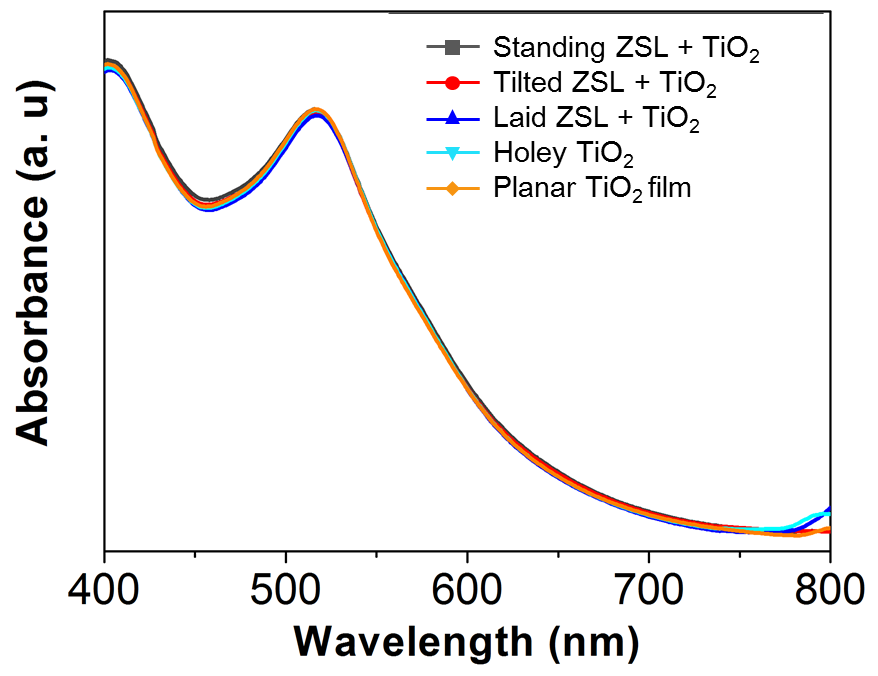


Figure S5. Absorbance spectra of dye molecules desorbed from different photoanodes.

Figure S5 is the absorbance spectra of N719 dye molecules that were desorbed from the five different photoanodes, showing that the amount of dye molecules is nearly identical in the five photoanodes.


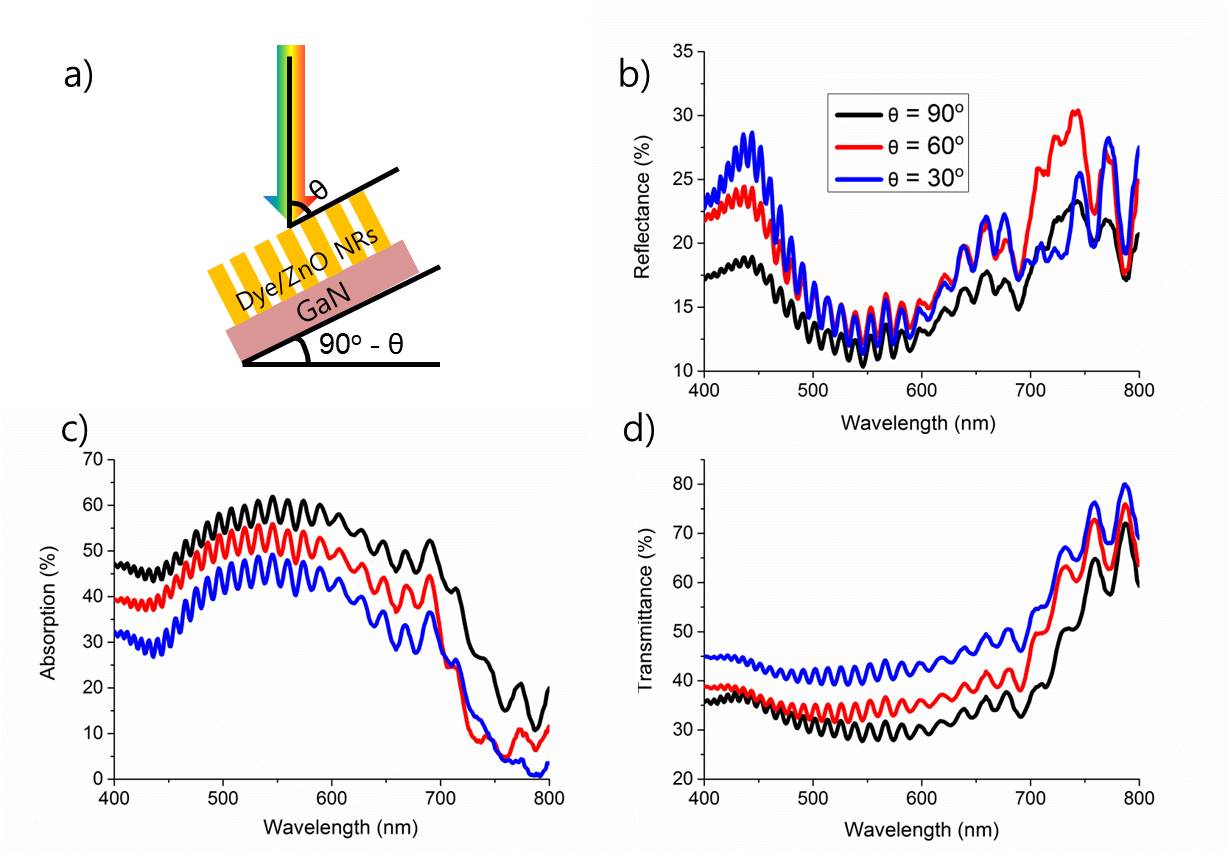


**Figure S6. Optical spectra of dye-loaded vertical ZnO NRs array at different incident angles.**

Vertically aligned ZnO NRs array was grown on a GaN substrate by hydrothermal method, and it was adsorbed with dye molecules. This sample was illuminated by halogen light source (white light) with three different incident angles (30^o^, 60^o^ and 90^o^) as shown in Figure S6(a). The base line was measured with a GaN substrate without the ZnO NRs, tilted at the corresponding angle of (90-θ) ^o^. Figure S6(b) and (c) are the reflectance and absorption spectra at different incident angles, respectively. Light is scattered stronger with decreasing angle but, less light is absorbed. These phenomena are the same as the results with the dye-adsorbed ZnO NRs, which were embedded into the TiO_2_ layer with different orientations in the photoanode, as described in the manuscript. More efficient light utilization can be achieved by absorbing and scattering more light in the photoanode. Therefore, a lower transmittance of the photoanode is required for the maximum light utilization. As shown in transmittance results of Figure S6 (d), T = 100 (%) – R – A, the vertical dye-adsorbed ZnO NRs sample reveals the lowest transmittance.
